# Anaemia, Iron Deficiency, and Functional Outcomes in Rural Older Indians: Insights from the Odisha Tribal Family Health Survey

**DOI:** 10.1016/j.jnha.2025.100730

**Published:** 2025-11-20

**Authors:** Jaya Singh Kshatri, Kavitha AK, Haimanti Bhattacharya, Sanghamitra Pati

**Affiliations:** a ICMR-Regional Medical Research Centre (Dept. of Health Research, Ministry of Health & Family Welfare, Govt. of India), Chandrasekharpur, Bhubaneswar 751023, India; b Academy of Scientific and Innovative Research (AcSIR), Ghaziabad, Uttar Pradesh, India; c Indian Council of Medical Research Centre (Dept. of Health Research, Ministry of Health & Family Welfare, Govt. of India), New Delhi, India

## 1 Introduction

Anaemia in older people has been a concern highlighted in this journal frequently [1]. Anaemia has been associated with higher mortality, frailty, decreased muscle strength, and functional decline with reports from China, and other Asian countries among others reflecting the significance of this health challenge in older people [1,2]. Extensive research has linked anaemia to reduced functional capacity, increased frailty, cognitive decline, and heightened mortality risk among older adults [3].

In this context, in a country like India with a rapidly expanding population of older people, anaemia is a major public health concern [4]. India’s Anaemia Mukt Bharat (“Anaemia Free India” programme) only targets women of reproductive age and children, overlooking older adults, despite their high vulnerability to iron deficiency–related anaemia [5].

In this correspondence, we analyse data from the Odisha Tribal Family Health Survey (OTFHS) with an aim to describe the prevalence and determinants of anaemia among older indigenous people. We also briefly present modelling-based projections on the benefit of iron supplementation on quality of life and disability-free years in this population.

## 2 Methods

This is an analysis from a large-scale sample survey among indigenous people of Odisha, India, the OTFHS, conducted between 2022–2023, using a multistage, stratified, probability-proportional-to-size (PPS) cluster sampling design [6]. We recruited participants aged ≥60 years who had lived in surveyed villages for at least six months.

Data on anaemia and its severity were collected and defined using WHO criteria. Predictors included demographic, nutritional, and functional markers (BMI, frailty), clinical comorbidities, and iron profile parameters. Multivariable logistic regression identified independent predictors of anaemia presence and severity, while a multinomial regression modelled anaemia aetiology (IDA, ACD, unclassified). Model covariates included age, sex, education, wealth quintile, BMI, comorbidities, frailty status, and biochemical indices.

Missing data (<10%) were imputed using mean value replacement for continuous variables, otherwise, complete-case analyses were used. Markov models were used to explore possible pathways of transition following iron supplementation [7].

## 3 Results

A total of 3,221 rural tribal adults aged ≥60 years were included in the final analysis; overall anaemia prevalence was 80.8%. Key demographic and clinical characteristics are summarized in Table 1. Serum iron, albumin, transferrin saturation, BMI, and EQ5D scores progressively declined with anaemia severity, while frailty and ADL dependence increased markedly. Severe anaemia showed lowest iron (60.8 μg/dL), BMI (18.2), and quality-of-life (0.829), with 21.5% ADL dependence, underscoring the strong nutritional linkage. Among anaemic individuals, 7.3% had iron deficiency anaemia (IDA), 27.1% anaemia of chronic disease (ACD) and 44.7% unclassified anaemia.

**Table 1:** Distribution of risk factors by anaemia severity in older adults.

Risk Factor	Anaemia				p-value
	None (n = 670)	Mild (n = 883)	Moderate (n = 1,375)	Severe (n = 288)	
**Age Group**					<0.001
<70 years	461 (69%)	607 (69%)	849 (62%)	156 (54%)	
70–79 years	171 (26%)	225 (25%)	440 (32%)	93 (32%)	
≥80 years	38 (5.7%)	51 (5.8%)	86 (6.3%)	39 (14%)	
**Sex**					0.093
Male	140 (21%)	539 (61%)	860 (63%)	191 (66%)	
Female	530 (79%)	344 (39%)	515 (37%)	97 (34%)	
**Wealth Quintile**					0.980
1 (Lowest)	112 (17%)	210 (24%)	298 (22%)	52 (18%)	
2	130 (19%)	156 (18%)	282 (21%)	57 (20%)	
3	134 (20%)	156 (18%)	281 (20%)	66 (23%)	
4	130 (19%)	154 (17%)	234 (17%)	55 (19%)	
5 (Highest)	164 (24%)	207 (23%)	280 (20%)	58 (20%)	
**BMI Category**					<0.001
Below normal	198 (30%)	398 (45%)	624 (45%)	171 (59%)	
Normal	430 (64%)	436 (49%)	701 (51%)	110 (38%)	
Overweight	38 (5.7%)	40 (4.5%)	38 (2.8%)	7 (2.4%)	
Obese	4 (0.6%)	9 (1.0%)	12 (0.9%)	0 (0.0%)	
**Hypertension**					0.219
No	569 (85%)	744 (84%)	1,112 (81%)	235 (82%)	
Yes	77 (11%)	102 (12%)	207 (15%)	50 (17%)	
**Diabetes**					0.501
No	629 (94%)	827 (94%)	1,285 (93%)	278 (97%)	
Yes	16 (2.4%)	20 (2.3%)	36 (2.6%)	6 (2.1%)	
**eGFR <60**					<0.001
Absent	544 (81%)	711 (81%)	994 (72%)	140 (49%)	
Present	126 (19%)	172 (19%)	381 (28%)	148 (51%)	
**Frailty**					<0.001
Non-frail	64 (9.6%)	140 (16%)	161 (12%)	21 (7.3%)	
Frail	606 (90.4%)	743 (84%)	1,214 (88%)	267 (93%)	
**Multimorbidity**					<0.001
No (0–1 condition)	411 (47%)	411 (47%)	513 (37%)	48 (17%)	
Yes (≥2 conditions)	259 (53%)	472 (53%)	862 (63%)	240 (83%)	

**Note**: p-values from chi-square or Fisher’s exact test. Column values are n (%). BMI: Body Mass Index. eGFR: Estimated Glomerular Filtration rate.

**Fig. 1:**
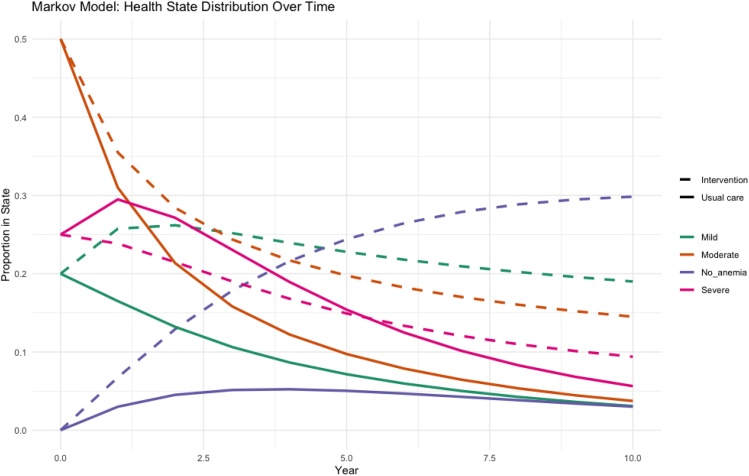
Modelling to show changes in anaemia states over time in an iron supplementation intervention scenario.

Regression models showed Anaemia to be associated with a modest reduction in quality of life. Additionally, age, CKD, and low wealth was associated with significantly reduced QoL, especially among those with frailty or comorbidities. Modelling of iron supplementation in older adults with IDA showed an improved EQ5D (by 0.055), yielding 5,512 Quality Adjusted Life Years (QALY) per 100,000 people. (See Fig. 1 for details) This was equivalent to 31 USD per QALY, which indicated excellent thresholds for possible cost-effectiveness.

## 4 Discussion

This study reveals an alarmingly high anaemia prevalence (>80%) among older tribal adults in rural Odisha, well above the national average of 68.3% and other international estimates previously reported here [4]. Moderate-to-severe anaemia predominates, indicating an urgent need for targeted action. Advancing age, male sex, low BMI, chronic kidney disease, hypoalbuminemia, and frailty independently predicted anaemia, aligning with global evidence linking malnutrition, multimorbidity, and chronic inflammation to its development [3,4].

Biomarker analyses showed that only 13.7% of cases were due to iron deficiency, while most were of mixed or unclassified origin, reflecting the complex and multifactorial nature of anaemia in older adults. Anaemia was strongly associated with poorer quality of life, greater frailty, and increased dependence in daily activities. Economic modelling indicated that iron supplementation for iron-deficient individuals is likely to be cost-effective (31 USD or ₹2,721/QALY gained), suggesting significant potential for improving functional health and reducing disability [5].

However, current national initiatives like Anaemia Mukt Bharat overlook this population. Expanding screening and interventions to include adults over 60, especially in high-risk populations like indigenous groups, is essential. Future randomised trials to identify optimal supplementation strategies, dosing, and safety profiles are needed.

## CRediT authorship contribution statement

Concept and design: JSK and SP. Acquisition, analysis or interpretation of data: JSK, KAK and HB. Statistical analysis: JSK and KAK. Drafting of the manuscript: JSK and HB. Critical revision of the manuscript for important intellectual content: SP.

## Ethics approval and consent to participate

The OTFHS was approved by the Institutional Human Ethics Committee of ICMR-RMRC Bhubaneswar (Reference no: ICMR-RMRC/IHEC-2022/115; Dated: 02/05/2022). All participants provided written informed consent.

## Funding

The study was funded by the Scheduled Castes and Scheduled Tribes Research and Training Institute (SCSTRTI), Odisha, India.

## Data availability

Data will be uploaded to a central repository of ICMR and will be made available upon request on the portal.

## Declaration of competing interest

The authors declare no competing interests.
